# Cannabinoid Receptor 1 and Fatty Acid Amide Hydrolase Contribute to Operant Sensation Seeking in Mice

**DOI:** 10.3390/ijms18081635

**Published:** 2017-07-27

**Authors:** Alexander I. Helfand, Christopher M. Olsen, Cecilia J. Hillard

**Affiliations:** Department of Pharmacology and Toxicology and Neuroscience Research Center, Medical College of Wisconsin, 8701 Watertown Plank Road, Milwaukee, WI 53226, USA; ahelfand@mcw.edu

**Keywords:** knock out, reward, motivation, novelty seeking, behavior

## Abstract

A large body of evidence in humans and preclinical models supports a role for the endocannabinoid system in the proper execution of motivated or goal-directed behaviors. Operant sensation seeking (OSS) is a task that uses varied sensory stimuli as a reinforcer to maintain operant responding in mice. The purpose of the studies in this report was to begin to explore the role of endocannabinoid signaling in OSS utilizing cannabinoid receptor 1 (CB1R) and fatty acid amide hydrolase (FAAH) knock out mice. Compared to wild type littermate controls, CB1R knock out mice exhibited significantly fewer active responses and earned significantly fewer reinforcers in fixed ratio and progressive ratio schedules. On the other hand, FAAH knock out mice exhibited increased active responses and earned more reinforcers than wild type littermates in fixed ratio but not progressive ratio schedules. These findings support the role of endocannabinoid signaling in motivated behaviors and also expand our understanding of the signaling processes involved in OSS.

## 1. Introduction

Considerable evidence supports a vital influence of the endocannabinoid system (ECS) in the brain in motivated behavior through its modulation of reward circuits; roles in associative learning, memory extinction and executive function; and a role in maintaining emotional homeostasis [1,2]. The primary receptor of the ECS found in the brain is the subtype 1 of the cannabinoid receptor (CB1R). The CB1R is a G protein-coupled receptor that is expressed throughout the human brain, particularly in regions involved in reward, emotional regulation and memory [3]. The endogenous ligands for the CB1R include two well-characterized arachidonates, 2-arachidonoylglycerol (2-AG) and *N*-arachidonoylethanolamine (AEA) [4]. Endocannabinoids are “made on demand” from lipid precursors in a receptor-dependent fashion, and act as retrograde signaling molecules [4]. Termination of AEA and 2-AG-mediated signaling involves hydrolysis; in particular, AEA in the brain is hydrolyzed almost exclusively by fatty acid amide hydrolase (FAAH) [5].

The contribution of the ECS to risk for addiction in humans can be inferred from candidate gene studies. Genetic variants in both the gene for the CB1R (*cnr1*) and for FAAH (*faah*) have been associated with dysregulation of reward-driven processes. For example, the number of repeats of an AAT-triplet microsatellite in the 3′-untranslated region of *cnr1* is associated with substance abuse disorders in Caucasians [6]. A silent single nucleotide polymorphism (SNP) in the coding region of *cnr1* (rs1049353) has been associated with enhanced withdrawal delirium in alcoholics [7] and enhanced impulsivity in individuals with longstanding substance dependence [8]. This SNP is also associated with anhedonia in individuals exposed to early life physical abuse [9], suggesting that the CB1R is involved in fundamental reward processing. Similarly, humans treated with the CB1R antagonist, rimonabant, exhibited increased depressive symptoms [10], reflecting a loss of hedonic drive. A highly investigated SNP in *faah* (rs324420) that is thought to result in reduced FAAH activity [11,12] has been associated with polydrug abuse [11] and obesity [13], data which also support a link between ECS and reward-motivated behaviors. Although the data are not completely consistent [2], there is support in these studies for the hypothesis that activation of CB1R signaling by AEA is associated with increased risk for drug dependence.

Preclinical studies support roles for CB1R signaling and FAAH activity in motivated behavior. Pharmacological or genetic inhibition of CB1R signaling reduces operant responding for palatable food [14] and conditioned place preference (CPP) for social interaction and palatable food [15]. On the other hand, inhibition of FAAH-mediated AEA catabolism increases nicotine-induced CPP [16] and increases social play behavior in adolescent rats, a highly rewarding behavior [17]. Injections of the endocannabinoid AEA into the nucleus accumbens increase the frequency of positive reactions to tasting sugar in a dose-dependent manner [18]. Oxytocin-dependent social reward also requires AEA-dependent CB1R signaling [19]. In spite of these and other studies, there is much that we do not understand about the roles of the ECS in brain processes involved in the formation and execution of operant behaviors that are driven by rewarding stimuli.

Operant sensation seeking (OSS) is a method for the assessment of non-drug rewarded behavior that involves the self-administration of varied sensory stimulation [20,21,22]. The model was developed from studies demonstrating that several species will acquire and perform operant responses to obtain visual and/or auditory stimuli [23,24,25]. OSS is sensitive to disruption of dopaminergic signaling [20] and requires type 5 metabotropic glutamate receptors (mGluR5) [26,27,28,29]. Thus, OSS appears to require elements of the reward pathway that are also engaged by psychostimulant self-administration [30,31], but is unique in that the self-administration of novel sensory stimuli does not require a pharmacological reinforcer. Unlike food self-administration, disruption of dopamine D1 receptor or mGluR5 receptor signaling impairs OSS [20,26,27,28]. Thus, OSS is a unique non-drug self-administration procedure that models aspects of the link between novelty seeking and psychostimulant addiction [32,33,34].

Due to its influence on motivated behaviors, we hypothesize that loss of CB1R function will result in diminished performance in the OSS task. Conversely, we hypothesize that augmentation of the ECS will result in enhanced performance in the OSS task. We tested these hypotheses using CB1R and FAAH knockout mice, respectively.

## 2. Results

### 2.1. Cannabinoid Receptor 1 (CB1R)

**Figure 1:**
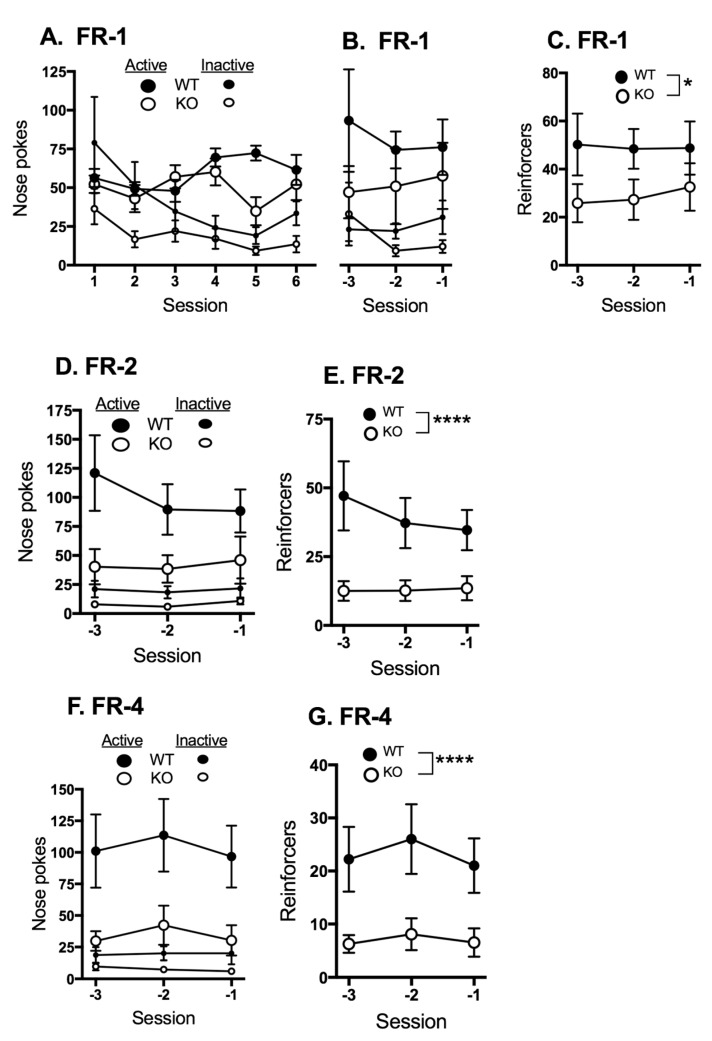
Comparison of wild type (WT) and CB1R knock out (CB1KO) active and inactive nose-poke responses and reinforcers earned during fixed ratio (FR) schedules. Mice (9 WT and 12 CB1KO) were placed into operant chambers with two nose-poke ports for 2 h daily for 6 consecutive days per week. Responses in one of the nose-poke ports triggered a combination of visual and auditory responses, the other did not. Mice progressed through three FR schedules in sequence. Responses in the active port are indicated with large circles, inactive with small circles. Filled circles represent data from WT mice, open circles from CB1KO mice. Mean values are shown, vertical lines represent standard error of the mean (SEM). Negative numbers in panels B–G denote the final three sessions prior to the session in which criteria were met for promotion of each animal to the next schedule. (**A**) Mean nose-poke responses in the active and inactive ports during the first six sessions of FR-1; (**B**) Mean nose-poke responses in the active and inactive ports during the final three sessions of FR-1; (**C**) Mean reinforcers earned during the final three sessions of FR-1; (**D**) Mean nose-poke responses in the active and inactive ports during the last three sessions of FR-2; (**E**) Mean reinforcers earned during the last three sessions of FR-2; (**F**) Mean nose-poke responses in the active and inactive ports during the last three sessions of FR-4; (**G**) Mean reinforcers earned during the last three sessions of FR-4. * *p* < 0.05, **** *p* < 0.0001.

Nose-pokes into the active and inactive ports for the first six sessions of fixed ratio-1 (FR-1) demonstrate that both genotypes progress to preference for the active port (Figure 1A). Active and inactive responding during the last three sessions before advancement to FR-2 for those mice that acquired the task are shown in Figure 1B. Two-way ANOVA of these data demonstrate a significant main effect of response type (i.e., active versus inactive) for both wild type (WT) (F_1,48_ = 18.02, *p* = 0.0002) and CB1R knock out (CB1KO) (F_1,66_ = 5.75, *p* = 0.0194). The average number of responses of both WT and CB1KO mice were greater on the active compared to the inactive port. The number of reinforcers that were triggered in response to the nose-pokes during the last three sessions of FR-1 are shown in Figure 1C. The WT mice accumulated more reinforcers on average than the CB1KO mice; two-way ANOVA indicates a significant main effect of genotype (F_1,57_ = 6.6, *p* = 0.013) without significant main effect of session or a significant interaction.

The number of nose-pokes into the active and inactive ports (Figure 1D) and reinforcers delivered (Figure 1E) for the last three sessions of FR-2 are shown. There was a significant effect of genotype on responses in both the active (F_1,57_ = 12.38, *p* = 0.0009) and inactive (F_1,57_ = 9.61, *p* = 0.003) ports; with WT mice exhibiting a greater number of responses compared to the CB1KO mice. The number of reinforcers delivered to the WT mice was very significantly greater than to the CB1KO mice (F_1,57_ = 23.03, *p* < 0.0001). Responding was stable across the three sessions; session as a main factor had no significant effect on responses or reinforcer delivery.

The number of nose-pokes into the active and inactive ports and reinforcers delivered for the last three sessions of FR-4 are shown (Figure 1F,G). Similar to the results of FR-2, both groups of mice exhibit stable responding over this period. Genotype significantly affected responding on both the active (F_1,57_ = 19.17, *p* < 0.0001) and inactive (F_1,57_ = 10.26, *p* = 0.0022) ports, with CB1KO mice exhibiting significantly lower activity at both ports compared to WT. Genotype significantly affected reinforcers earned (F_1,57_ = 22.4, *p* < 0.0001); the CB1KO mice received fewer reinforcers than the WT mice.

**Figure 2:**
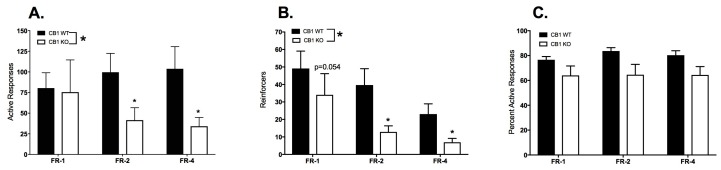
Comparison of FR-1, 2 and 4 responses and reinforcers earned in WT (*n* = 9; filled bars) and CB1KO (*n* = 12; open bars) mice. Bars represent mean ± SEM. (**A**) Mean responses in the active port; (**B**) Mean reinforcers earned; (**C**) Mean percent of total responses made in the active port. * *p* < 0.05.

The behavioral responses and reinforcers delivered during the last three sessions of the FR schedules are compared in Figure 2. The numbers of responses in the active port (Figure 2A) are not normally distributed and were converted to ranks for statistical analysis. Two-way ANOVA of the ranked data demonstrate significant main effects of genotype (F_1,19_ = 5.705, *p* = 0.0275), schedule (F_3,57_ = 46.64, *p* < 0.0001) and a significant interaction (F_3,57_ = 3.503, *p* = 0.0210). Post hoc tests revealed that CB1KO animals performed significantly fewer nose-pokes in the active port in the FR-2 and FR-4 conditions; *p* values adjusted for multiple comparisons (Holm-Sidak) were 0.0191 and 0.0179, respectively.

The numbers of reinforcers earned during the FR sessions (Figure 2B) are not normally distributed and were converted to ranks before being compared. Two-way ANOVA demonstrated a significant effect of genotype on reinforcers (F_1,19_) = 6.52, *p* = 0.019). There was a highly significant effect of schedule on these data (F_2,38_ = 17.49, *p* < 0.0001). Although the interaction was not significant (F_2,38_ = 0.52; *p* = 0.60), this was a planned comparison so post hoc tests were carried out. Holm-Sidak’s corrected multiple comparisons found that the CB1KO animals earned significantly fewer reinforcers than WT during FR-2 (*t*_57_ = 2.62, *p* = 0.033) and FR-4 (*t*_57_ = 2.44, *p* = 0.035), and trended to fewer reinforcers in FR-1 (*t*_57_ = 1.97, *p* = 0.054).

The percent of total responses in the active port in the final three sessions of each FR schedule are shown in Figure 2C. This metric is an indicator of the animal’s accuracy in performing the behavioral task independent of overall activity. Two-way ANOVA of the data indicated a nearly significant effect of genotype on the percent active responses (F_1,19_ = 4.02, *p* = 0.059).

**Figure 3:**
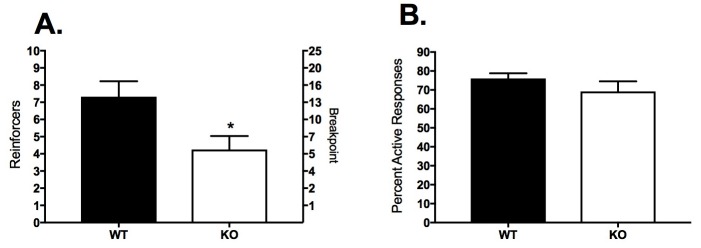
Responses and reinforcers earned during the progressive ratio (PR) schedule in WT (*n* = 9; filled bars) and CB1KO (*n* = 12; open bars) mice. Bars represent mean ± SEM. (**A**) Left *y*-axis depicts the reinforcers earned; right y-axis depicts the breakpoint associated with each reinforcer; (**B**) Mean percent of total responses made in the active port. * *p* < 0.05.

After FR-4, all subjects were advanced to progressive ratio (PR) for three sessions; the average responses and reinforcers across the 3 days are shown in Figure 3. The CB1KO mice received significantly fewer reinforcers and exhibited a lower breakpoint than the WT mice (Figure 3A; *t*_19_ = 2.57, *p* = 0.019). There was no significant difference between genotypes in the percent of responses that were in the active port (Figure 3B; *t*_19_ = 1.03, *p* = 0.316).

### 2.2. Fatty Acid Amide Hydrolase (FAAH)

**Figure 4:**
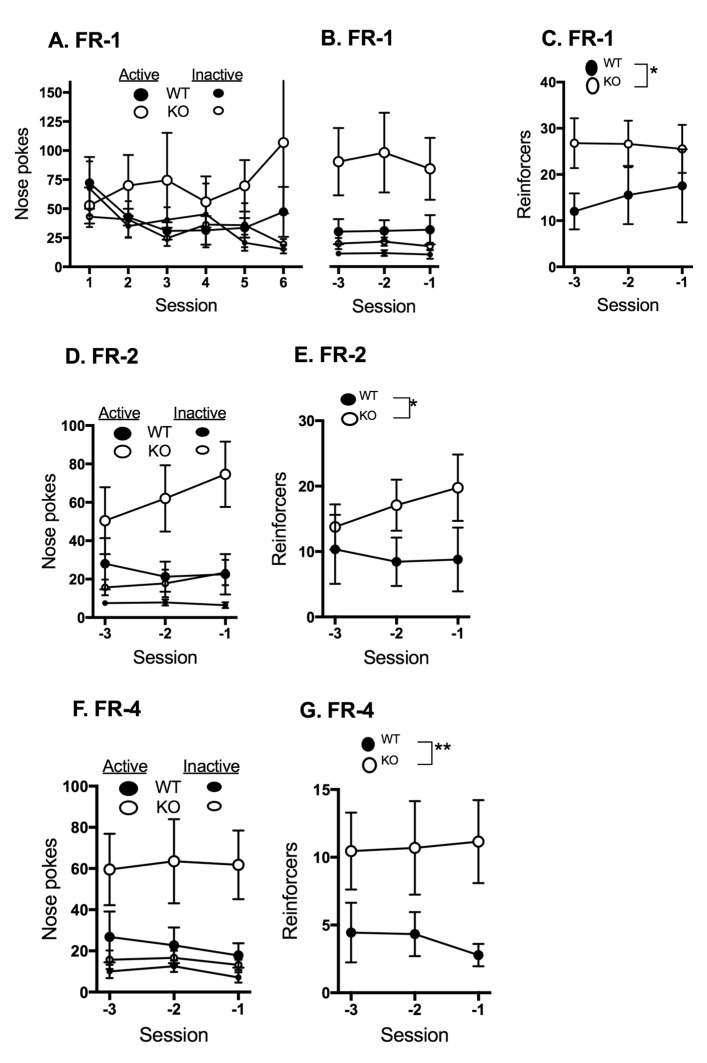
Comparison of WT and fatty acid amide hydrolase knock out (FAAHKO) active and inactive nose-poke responses and reinforcers earned during FR schedules. Mice (9 WT and 13 FAAHKO) were placed into operant chambers with two nose-poke ports for 2 h daily for 6 consecutive days per week. Responses in one of the nose-poke ports triggered a combination of visual and auditory responses, the other did not. Mice progressed through three FR schedules in sequence. Responses in the active port are indicated with large circles, inactive with small circles. Filled circles represent data from WT mice, open circles from FAAHKO mice. Mean values are shown; vertical lines represent SEM. Negative numbers in panels B–G denote the final three sessions prior to the session in which criteria were met for promotion of each animal to the next schedule. (**A**) Mean nose-poke responses in the active and inactive ports during the first six sessions of FR-1; (**B**) Mean nose-poke responses in the active and inactive ports during the final three sessions of FR-1; (**C**) Mean reinforcers earned during the final three sessions of FR-1; (**D**) Mean nose-poke responses in the active and inactive ports during the last three sessions of FR-2; (**E**) Mean reinforcers earned during the last three sessions of FR-2; (**F**) Mean nose-poke responses in the active and inactive ports during the last three sessions of FR-4; (**G**) Mean reinforcers earned during the last three sessions of FR-4. * *p* < 0.05, ** *p* < 0.01.

Acquisition of the OSS operant task in FAAH knock out (FAAHKO) and WT littermates during the first 6 sessions of FR-1 is shown in Figure 4A. It is noteworthy that the WT controls in the FAAHKO breeding colony exhibit significantly lower numbers of responses than the WT controls in the CB1KO breeding colony. Both genotypes exhibit stable responding in the last 3 sessions of FR-1 (Figure 4B); two-way ANOVA demonstrates significant differences in responding in the active and inactive ports for both WT (F_1,51_ = 10.01, *p* = 0.0026) and FAAHKO (F_1,72_ = 16.44, *p* < 0.0001) mice. There was a significant effect of genotype on the number of reinforcers earned (Figure 4C; F_1,60_ = 5.736, *p* = 0.0198), with the FAAHKO mice receiving a greater number of reinforcers.

Two-way ANOVA demonstrates a significant effect of response type in both the FAAHKO (F_1,72_ = 16.88, *p* = 0.0001) and WT (F_1,48_ = 6.96, *p* = 0.0112) mice responding under the FR-2 schedule (Figure 4D). There is a significant main effect of genotype on the numbers of reinforcers earned (Figure 4E; F_1,60_ = 4.34, *p* = 0.0413), with the FAAHKO mice earning more reinforcers than the WT mice. There were no significant main effects of session on either responses or reinforcers, indicating that the responses were stable over these FR-2 sessions.

Under FR-4 conditions, responses (Figure 4F) and reinforcer delivery (Figure 4G) remained stable, with significant effects of genotype on responses in the active port (F_1,60_ = 8.45, *p* = 0.0051) and reinforcers earned (F_1,60_ = 8.85, *p* = 0.0042). The FAAHKO mice had more nose-pokes in the active port and more reinforcers earned than the WT.

**Figure 5:**
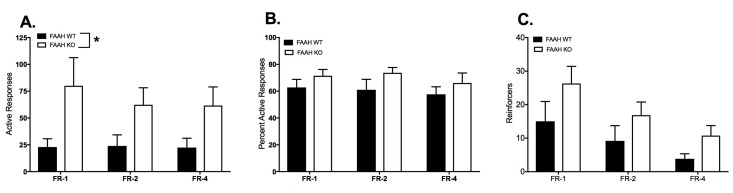
Comparison of FR-1-4 responses and reinforcers earned in WT (*n* = 9; filled bars) and FAAHKO (*n* = 13; open bars) mice. Bars represent mean ± SEM. (**A**) Mean responses in the active port; (**B**) Mean percent of total responses made in the active port; (**C**) Mean reinforcers earned. * *p* < 0.05.

Comparisons between WT and FAAHKO mice of the mean responses during the last three sessions across the three FR paradigms are presented in Figure 5. The active response data (Figure 5A) were not normally distributed and were converted to ranks. Two-way ANOVA of the ranked data demonstrates a significant effect of genotype on active responses (F_1,20_ = 5.63, *p* = 0.028). There was no significant main effect of schedule nor was there a significant interaction. Comparisons between WT and FAAHKO were planned; however, there were no significant differences between WT and FAAHKO mice in any schedule.

The percent of responses in the active port (Figure 5B) was not normally distributed and was converted to ranks prior to analysis. Two-way ANOVA of the ranked data demonstrated a slight trend toward a main effect of genotype (F_1,20_ = 3.07, *p* = 0.095). Schedule and interaction were not significant.

The numbers of reinforcers earned (Figure 5C) were not normally distributed and were converted to ranks prior to analysis. Two-way ANOVA of the ranked data revealed a very significant main effect of schedule (F_2,40_ = 29.11, *p* < 0.0001) and a trend toward a significant main effect of genotype (F_1,20_ = 4.265, *p* = 0.052). There were no significant differences between genotypes at each schedule in the planned post hoc analysis.

**Figure 6:**
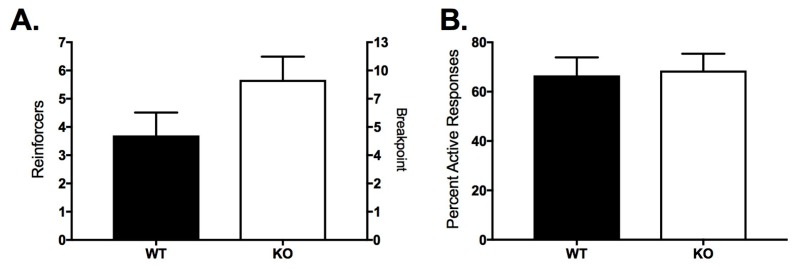
Responses and reinforcers earned during the PR schedule in WT (*n* = 9; filled bars) and FAAHKO (*n* = 13; open bars) mice. Bars represent mean ± SEM. (**A**) Left *y*-axis depicts the reinforcers earned; right *y*-axis depicts the breakpoint reached; (**B**) Mean percent of total responses made in the active port.

After FR-4, all subjects were advanced to PR for three sessions; the average responses and reinforcers across the 3 sessions are shown in Figure 6. There was no significant difference between the groups in reinforcers earned or in the breakpoint during the PR paradigm (Figure 6A; *t*_20_ = 1.64, *p* = 0.117). The percent of responses in the active port were not different between the genotypes (Figure 6B; Mann Whitney U = 55, *p* = 0.845).

## 3. Discussion

C57Bl/6J mice establish stable operant responding to receive visual and auditory stimuli, a process named “operant sensation seeking” [20,21,22,26]. In this study, we report that outbred male Institute for Cancer Research (ICR) mice also learn and perform OSS at response rates that are in the range of those seen in studies with C57Bl/6J mice. The first hypothesis of the present study was that loss of CB1R function would negatively impact OSS; our results support this hypothesis. Although the CB1KO mice are able to learn the task, they exhibit significantly fewer active responses in FR-2 and FR-4 and a significantly lower breakpoint in PR than WT littermates. As a result, they earn significantly fewer reinforcers in each of these paradigms as well. The reduction in responses in the active port was paralleled by a decrease in responding in the inactive port, providing evidence that CB1KO mice have learned the task but are not as motivated to respond. This conclusion is supported by the percent active response data, which indicate that both genotypes are responding in the active port at rates higher than chance. There was a trend to a reduction for the CB1KO compared to WT in percent active responses in the FR paradigms, but no difference in the PR paradigm, suggesting very little difference in this parameter between the genotypes in spite of the reduced overall responding of the CB1KO mice. Very similar effects were seen in CB1KO mice trained to nose-poke to receive Ensure^®^ (Abbot); CB1KO mice exhibit reduced responding in an FR-1 schedule in both active and inactive ports compared to WT mice [35]. CB1KO mice also respond significantly less well for sucrose under an FR-1 schedule [36], and for sucrose and Ensure^®^ under PR schedules [36]. CB1KO mice also exhibit reduced operant self-administration and CPP of ethanol, nicotine and opiates, but do not exhibit differences in stimulant-driven behaviors [2].

Comparison of OSS between FAAHKO and littermate WT controls demonstrates some support for our hypothesis that FAAHKO mice would exhibit increased OSS. FAAHKO mice received significantly more reinforcers in the last three sessions of FR-1, FR-2 and FR-4 compared to WT. The number of responses in the active port was significantly greater in the FR sessions without a difference in percent active responses. In contrast to the significant effects seen in the FR schedules, FAAHKO were not significantly different from WT littermates in reinforcers earned or breakpoint in the PR schedule. This suggests that the changes evoked by FAAH deletion are sufficient to increase responding under high ratios of reinforcer to response but not when the work required to obtain a reinforcer is increased. These findings are consistent with reports that FAAH deficiency enhances motivation for food [37] and increases operant self-administration of ethanol in alcohol-preferring rats [38]. FAAH inhibition also increases nicotine CPP in mice through a CB1R mechanism [39]. FAAHKO mice have normal 2-AG concentrations [5]**,** and recent data suggest that 2-AG-mediated signaling at CB1R promotes CPP for social interactions and high fat food [15], which suggests that elevation of AEA only activates a subset of CB1R signaling in the reward circuit. OSS is also sensitive to loss of mGluR5 signaling [26] and mGluR5 mobilizes endocannabinoid signaling in many brain regions [4], including the nucleus accumbens [40].

These studies did not examine the mechanisms or sites at which CB1R signaling regulates OSS; however, OSS requires intact dopaminergic signaling [20] and CB1R antagonists block the effects of multiple drugs of abuse to affect phasic dopamine release in the ventral striatum [41]. Conversely, exogenous treatment with CB1R agonists [42], including AEA [43], increases dopamine concentrations in the nucleus accumbens. CB1R signaling can regulate dopamine release through effects in the ventral striatum [42] and also modulates synaptic activity in the ventral tegmental area (VTA), and can thereby alter activation of VTA-accumbens projections [44,45,46].

There are several limitations to this study. Lifelong genetic deletion of the CB1R and FAAH could result in compensatory changes that could contribute to the changes seen here. In addition, we have not examined the role of the CB1R in the changes observed in the FAAHKO mice. Finally, there are differences in responses between the WT mice of the CB1R colony and the FAAH colony. We cannot account for these differences and assume that the outbred mice used as founders of the lines had a genetic or epigenetic difference that was inherited by the colony.

In summary, these data support the use of OSS to study the role of the CB1R and FAAH in operant behavior. They also expand on previous work [20,21,22] that suggests that reinforcing sensory stimuli, like other natural rewards, are sensitive to disruption of CB1R signaling.

## 4. Materials and Methods

### 4.1. Animals

Male ICR mice (ages 8–20 weeks old) were utilized in this study. Knock out and wild type (WT) mice were generated from heterozygous breeding pairs, and comparisons were made within littermates. The CB1R knock out (CB1KO) mice were generated on a 129/Svj background and backcrossed with outbred ICR WT mice for at least ten generations [46,47]. The FAAH knock out (FAAHKO) mice were initially generated on a 129/Svj background, backcrossed with C57BL/6 mice [5,48], then backcrossed with ICR mice for ten generations. Mice were genotyped at weaning by performing PCR on ear punches. Genotype was confirmed following completion of the protocol using fresh tissue samples taken from the animal’s ear immediately following euthanasia. PCR was conducted with FAAH and CB1 forward and reverse primers (Eurofins Genomics, Louisville, KY, USA). Primer sequences for the CB1 animals were: 5′-TATCTAGAGGCTGCGCAGTGCCTTC-3′ (WT forward), 5′-CCCTCTGCTTGCGATCATGGTGTAT-3′ (common reverse), and 5′-GGGCCAGCTCATTCCTCCCACTCAT-3′ (KO reverse). Primer sequences for the FAAH animals were: 5′-TAACTAGGCAGTCTGACTCTAG-3′ (WT forward), 5′-ACTCAAGGTCAGCCTGAAACC-3′ (common reverse), and 5′-TTTGTCACGTCCTGCACGACG-3′ (KO reverse).

Mice were housed in an AAALAC-approved animal care facility on a reverse light cycle (lights off 00730–1930) and experiments were conducted between 1500 and 1700. Food and water were provided ad libitum during all experiments. Mice were group housed for the duration of the experiment and handled by the experimenter for a minimum of two days prior to initiating experiments. Mice were weighed daily following each session. All of the procedures utilized were approved by the Institutional Animal Care and Use Committee of the Medical College of Wisconsin (AUA2698, 03/05/2015).

### 4.2. Operant Sensation Seeking (OSS)

Based upon previous data using C57BL/6J mice [26], we determined from power analysis that 9 mice per group would be sufficient to detect a 30% change in reinforcers earned with a power of 0.8 and alpha of 0.05. There are unequal numbers of mice in each group because we used all of the mice available in the litters that were studied. Mice were placed into operant chambers (Med Associates, St. Albans, VT, USA) equipped with two recessed ports for nose-pokes 2.2 cm above the grid floor as described [49]. Prior to the start of an experimental session, chambers were cleaned with 30% ethanol. Interaction with the active nose-poke port resulted in a compound visual/auditory stimulus according to the reinforcement schedule described in Table 1. The visual stimulus was presented using yellow light emitting diode (LED) lamps mounted 2 cm above the ports and consisted of a 2–8 s randomized light pattern as described [20,21,22]. The auditory stimulus was generated by activation of an infusion pump located adjacent to the chamber (no infusion was made). These stimuli are subsequently referred to as the reinforcer. The nose-poke ports were inactive while the reinforcer was being provided. Interactions with the incorrect port were recorded but did not result in any consequence. The experimenter was blinded to the genotype of the mice, and assignment of the active nose-poke port to the right or left side of the chamber was randomly chosen and counterbalanced.

Mice underwent daily 2-h sessions until promotion criteria were met (Table 1). Sessions occurred on 6 consecutive days, alternating with a 1 day break. Animals failing to meet promotion criteria described below at the conclusion of any reinforcement schedule were promoted to the next schedule regardless of behavior. Three fixed ratio (FR) schedules were utilized: FR-1 provided reinforcer after one response in the active port, FR-2 required two active responses, and FR-4 required four. At the conclusion of a particular reward schedule, animals which had not yet met criteria were promoted to the next schedule regardless of performance.

After six sessions of FR-4 (or sooner if the animal met promotion criteria described in Table 1), the animal entered the progressive ratio (PR) reinforcement schedule. PR consisted of progressively increasing numbers of correct nose-pokes required to trigger a reinforcer. The ratio of active responses required to elicit reinforcer delivery was calculated as (5*e*^(reinforcers earned*0.18)^) − 5, rounded to the nearest integer [49,50]. The reinforcement schedule reset to 1 at the start of each PR session. The experiment concluded after three PR sessions.

For analysis, we determined the following: the number of responses in the active and inactive ports; the number of reinforcers delivered; total responses; and the ratio of responses to reinforcers in the PR paradigm.

### 4.3. Data Analysis

Data were compared using a two-way repeated measures ANOVA in cases where data followed a Gaussian distribution. Where the data did not fit a Gaussian distribution, data were converted into ranks prior to comparison using ANOVA. Shapiro-Wilk’s test was used to assess normality, and additional post hoc *t*-tests (Welch’s *t*-tests when unequal variances were found with Levene’s test) or Mann-Whitney U tests (depending on normality) were performed and corrected using Holm-Sidak’s stepwise method.

## Figures and Tables

**Table 1:** Parameters for fixed ratio (FR) paradigms.

Schedule	Duration of Schedule	Activity Criteria	Correct Behavioral Acquisition	Deviation Criteria and Stable Responding
FR-1	At least 6 sessions, no more than 12	At least 20 reinforcers earned	2:1 active:inactive response ratio	Reinforcers do not deviate beyond 20% of a 3 day moving average
FR-2	At least 3 sessions, no more than 6	At least 15 reinforcers earned		
FR-4	At least 3 sessions, no more than 6	At least 15 reinforcers earned		

## Abbreviations

2-AG	2-Arachidonoylglycerol
AEA	*N*-arachidonoylethanolamine
CB1KO	Cannabinoid type 1 receptor knock out
CB1R	Type 1 cannabinoid receptor
ECS	Endocannabinoid signaling
FAAH	Fatty acid amide hydrolase
FAAHKO	Fatty acid amide hydrolase knock out
FR	Fixed ratio
mGluR5	Metabotropic glutamate type 5
OSS	Operant sensation seeking
PR	Progressive ratio
SNP	Single nucleotide polymorphism
VTA	Ventral tegmental area
WT	Wild type
